# A common variant of LDL receptor related protein 2 (*LRP2*) gene is associated with gout susceptibility: a meta-analysis in a Japanese population

**DOI:** 10.1007/s13577-019-00318-5

**Published:** 2020-01-24

**Authors:** Airi Akashi, Akiyoshi Nakayama, Yoichiro Kamatani, Toshihide Higashino, Seiko Shimizu, Yusuke Kawamura, Misaki Imoto, Mariko Naito, Asahi Hishida, Makoto Kawaguchi, Mikiya Takao, Michinori Matsuo, Tappei Takada, Kimiyoshi Ichida, Hiroshi Ooyama, Nariyoshi Shinomiya, Hirotaka Matsuo

**Affiliations:** 1 Department of Integrative Physiology and Bio-Nano Medicine, National Defense Medical College, 3-2 Namiki, Tokorozawa, Saitama 359-8513 Japan; 2 Laboratory for Statistical Analysis, RIKEN Center for Integrative Medical Science, Yokohama, Japan; 3 Graduate School of Frontier Sciences, The University of Tokyo, Tokyo, Japan; 4 Department of Preventive Medicine, Nagoya University Graduate School of Medicine, Nagoya, Aichi Japan; 5 Department of Oral Epidemiology, Hiroshima University Graduate School of Biomedical and Health Sciences, Hiroshima, Japan; 6 Department of Food and Nutrition, Faculty of Home Economics, Kyoto Women’s University, Kyoto, Japan; 7 Department of Pharmacy, The University of Tokyo Hospital, Tokyo, Japan; 8 Department of Pathophysiology, Tokyo University of Pharmacy and Life Sciences, Hachioji, Tokyo Japan; 9 Ryougoku East Gate Clinic, Tokyo, Japan

**Keywords:** Uric acid, Gout, Hyperuricemia, LRP2, Single nucleotide polymorphism (SNP)

## Abstract

Gout, which results from elevated serum uric acid (SUA), is a common form of arthritis that is induced by urate crystals. A single nucleotide polymorphism, rs2544390, of LDL receptor related protein 2 (*LRP2/Megalin*), has previously been reported to be associated with SUA by a genome-wide association study in a Japanese population. However, it was controversial as to whether rs2544390 is associated with gout in a Japanese population, since previous studies with Japanese populations have reported an association between gout and rs2544390 both with and without significance. This prompted us to investigate the association between gout and rs2544390 of *LRP2.* Using 1208 clinically diagnosed gout patients and 1223 controls in a Japanese male population, our results showed that while rs2544390 did not show a significant association with gout susceptibility in the present study (*p* = 0.0793, odds ratio [OR] with 95% confidential interval [CI] 1.11 [0.99–1.24]). However, a meta-analysis using previous studies on Japanese populations revealed a significant association with gout (*p*_meta_ = 0.0314, OR with 95% CI 1.09 [1.01–1.18]). We have therefore for the first time confirmed a positive association between rs2544390 and gout with only a Japanese male population. Our study provides clues to a better understanding of the pathogenesis of gout and has the potential to lead to novel therapeutic strategies against gout using LRP2 as a molecular target.

## Introduction

Gout is a common disease that results from an increase in serum uric acid (SUA), which can lead to renal failure, hypertension, and cardiovascular disease [1, 2]. rs2544390, a single nucleotide polymorphism (SNP) in LDL receptor related protein 2 (*LRP2*, also known as *Megalin*), was found to have an association with SUA in a genome-wide association study (GWAS) with 8868 Japanese [3]. However, the association between rs2544390 and gout remained to be clarified, because some studies, including ours, have reported no association [4, 5], while others have revealed a significant association [5–7]. In this study, we investigated a further association between gout and rs2544390 with clinically diagnosed gout patients and controls, and performed a meta-analysis based on past Japanese population studies [4, 6].

## Methods

### Patients and controls

1208 male Japanese patients were recruited from outpatients at Ryougoku East Gate Clinic (Tokyo, Japan). All these subjects had been diagnosed with primary gout according to the criteria established by the American College of Rheumatology [8]. As the control group, 1223 Japanese males without a history of gout or hyperuricemia (SUA levels > 7.0 mg/dL) were selected from participants in the Shizuoka area in the Japan Multi-Institutional Collaborative Cohort Study (J-MICC Study) [9]. The mean age and standard deviation of cases and controls were 45.4 ± 10.4 and 53.1 ± 8.8 years, respectively, and their mean body mass index was 25.4 ± 3.7 and 23.3 ± 2.7 kg/m^2^, respectively.

### Genetic and statistical analyses

Genomic DNA was extracted from whole peripheral blood [10]. Genotyping of *LRP2* polymorphism (rs2544390) was performed using a TaqMan assay (Custom TaqMan MGB, Applied Biosystems) with a Lightcycler 480 (Roche Diagnostics) as previously described [4]. To confirm their genotypes, more than 25 samples were subjected to direct sequencing with the following primers: for rs2544390, forward 5′-CTGTCTGAGACCATGACACAG-3′, and reverse 5′-CCTCACCTGTCATTGTCTTG-3′. DNA sequencing analysis was performed with a 3130xl Genetic Analyzer (Applied Biosystems) [11]. For the calculations in the statistical analyses, we used SPSS v.22.0J (IBM Japan Inc., Tokyo, Japan) and R (version 3.1.1) [12] including a meta-package [13]. The Chi-square test was used for the association and Hardy–Weinberg equilibrium analyses. A *p* value of < 0.05 was regarded as statistically significant.

## Results

Table 1 shows the genotyping results of rs2544390 for 1208 gout cases and 1223 controls. The genotyping call rate for this SNP was more than 98%. In the control group, this SNP was in Hardy–Weinberg equilibrium (*p* > 0.05), which suggested no mistyping.

**Table 1:** Association between gout and *LRP2* rs2544390 polymorphism

	Genotype			MAF^a^	Allele frequency mode	
	C/C	C/T	T/T		*p* value^b^	OR (95% CI)
Case	291	587	330	0.516	0.0793	1.11 (0.99–1.24)
Control	312	608	290	0.491		

*MAF* minor allele frequency, *OR* odds ratio, *CI* confidence interval

^a^T: minor allele

^b^Chi-square test of rs2544390 polymorphism

**Fig. 1:**
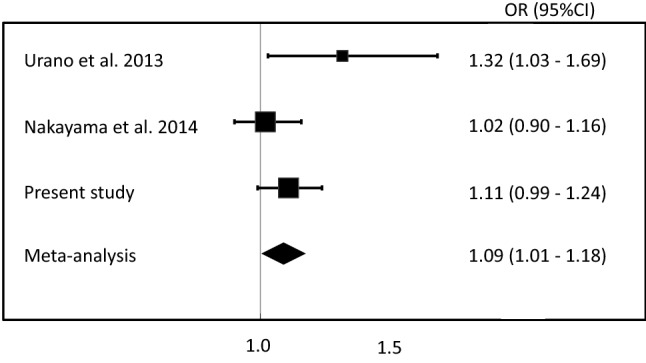
A meta-analysis of rs2544390 of *LRP2* for gout in the Japanese male population. The meta-analysis was conducted using the present study and two previous studies of Japanese male populations (Urano et al. [3] and Nakayama et al. [5]). The OR in the meta-analysis was 1.09 (95% CI 1.01–1.18) and was statistically significant (*p*_meta_ = 0.0314), indicating a significant association between gout and the *LRP2* gene. *OR* odds ratio, *CI* confidence interval

As in our previous study [4], rs2544390 did not show a significant association with gout susceptibility (*p* = 0.0793, odds ratio [OR] with 95% confidential interval [CI] 1.11 [0.99–1.24]: Table 1). The frequency of the minor risk allele, which in this study was the T allele of rs2544390, in the gout cases (51.6%) was higher than in the controls (49.1%). However, a meta-analysis including previous studies [4, 6] with a Japanese population showed a significant association with gout (*p*_meta_ = 0.0314, OR with 95% CI 1.09 [1.01–1.18]; Fig. 1).

## Discussion

We confirmed that rs2544390, an SNP of *LRP2*, has an association with gout in the Japanese population. LRP2 is a member of the low-density lipoprotein receptor [LDLR (MIM606945)] gene family and has been suggested to mediate the endocytosis of multiple ligands [14].

The association between rs2544390 and SUA was first identified by a GWAS of SUA in a Japanese population by Kamatani et al [3]; this was also confirmed by the recent genome-wide meta-analysis of SUA [15]. Since the association between *LRP2* and SUA was reported, several association studies for the Japanese population have been conducted, because gout is a consequence of hyperuricemia. From Japanese male populations, Urano et al. [6] reported a positive association between gout and rs2544390 (*p* = 0.0250, OR with 95% CI 1.32 [1.03–1.69]) in 153 gout cases and 532 controls, whereas Nakayama et al. [4] reported no association with gout (*p* = 0.758, OR with 95% CI 1.02 [0.90–1.16]) in 741 gout cases and 1302 controls. It therefore remained an open question as to whether rs2544390 has an association with gout in the Japanese population.

In other studies with non-Japanese populations, Dong et al. [7] showed a positive association with a Chinese male population of 483 gout cases and 389 controls (*p* = 0.020, OR with 95% CI 1.26 [1.03–1.53]). Furthermore, their meta-analysis with the results of Nakayama et al. [4] and Urano et al. [6] revealed a positive association with gout (*p* = 0.019, OR with 95% CI 1.13 [1.02–1.24]) [7], which is consistent with the conclusions of our present study. Another study from New Zealand by Rasheed et al. [5] with 1431 gout cases and 1205 controls showed a negative association between rs2544390 and gout of all the participants including European Caucasian (*p *= 0.360, OR with 95% CI 1.06 [0.94–1.18]), in contrast to a sub-analysis of only populations of Māori and Pacific ancestry (*p* = 0.0090, OR with 95% CI 1.20 [1.05–1.38]). These results suggest there to be ethnic differences for gout risk due to the *LRP2* variant and that Asia-Pacific populations should show a positive association between them.

Nakayama et al. [4], who previously reported no association between rs2544390 and gout, pointed out the need for further analysis with a greater number of samples to demonstrate any significant association between the *LRP2* variant in question and gout. While the present association study also showed no association, our meta-analysis with a greater number of samples showed a significant positive association between rs2544390 and gout in the Japanese population.

In the present study, we have confirmed for the first time a significant positive association between rs2544390 and gout, which has been shown in other populations by Dong et al. [7] and Rasheed et al. [5], in a Japanese population.

SUA levels are regulated by urate transporters that are expressed in the kidney and intestines. Recent GWASs of clinically defined gout have revealed associations between gout and urate transporter genes, such as *ABCG2/BCRP, URAT1/SLC22A12, GLUT9/SLC2A9, NPT1/SLC17A1* [16–19], all of which are expressed in the proximal tubular cells of the kidney [17]. Common variants of the urate excretion transporter *ABCG2* are known to significantly elevate gout/hyperuricemia susceptibility and are a major cause of early onset gout [20–22]. ABCG2 is expressed in the human intestine and is associated with intestinal urate excretion in addition to renal urate excretion [23, 24]. Furthermore, some association analyses have also shown associations between gout and urate transporter genes *URAT1* [25–27], *NPT1* [27–29], *OAT4/SLC22A11* [30] and *OAT10/SLC22A13* [31], and scaffold protein genes *PDZK1* [32] and *LRRC16A* [33], which bind to various transporters and form transportosomes with them. These genes, including urate transporter genes which are associated with SUA and gout/hyperuricemia, are mostly seen in the kidney, which is one of the urate-excreting organs. The *LRP2* gene is also strongly expressed in the kidney [14, 34, 35].

In conclusion, the association between a mutation of *LRP2* and gout is confirmed in addition to association with SUA. *LRP2* is thought to have a possible association with urate regulation and gout susceptibility by playing an important role in urate mobilization via endocytosis. The clarification of the association between *LRP2* and gout progression will lead to a better understanding of the molecular pathogenesis of gout and to novel therapeutic strategies against gout, using LRP2 as a molecular target. Our new insights into LRP2 have the potential to assist with the development of future personalized medicine.
